# Abundant circulating microRNAs in breast cancer patients fluctuate considerably during neoadjuvant chemotherapy

**DOI:** 10.3892/ol.2014.2188

**Published:** 2014-05-28

**Authors:** UGUR GEZER, SERKAN KESKIN, ABDULLAH İĞCI, MUSTAFA TÜKENMEZ, DUYGU TIRYAKIOĞLU, MERVE ÇETINKAYA, RIAN DIŞCI, NEJAT DALAY, YEŞIM ERALP

**Affiliations:** 1 Department of Basic Oncology, Oncology Institute, Istanbul University, Istanbul 34093, Turkey; 2 Department of Clinical Oncology, Oncology Institute, Istanbul University, Istanbul 34093, Turkey; 3 Department of General Surgery, Istanbul Medical Faculty, Istanbul University, Istanbul 34093, Turkey

**Keywords:** circulating microRNAs, neoadjuvant chemotherapy, breast cancer

## Abstract

Previous studies have revealed the aberrant expression of a number of microRNAs (miRNA/miRs) in the blood circulation of patients with breast cancer (BC). The aim of the present study was to assess the effect of neoadjuvant chemotherapy on the levels of a panel of BC-associated miRNAs, which are at relatively low (let-7, miR-10b, miR-34, miR-155, miR-200c and miR-205) or abundant (miR-21, miR-195 and miR-221) levels in the circulation. Patients with primary operable or locally advanced BC were enrolled in the study. The plasma levels of the miRNAs at baseline and at the fourth cycle of treatment were compared. Patients with stage II disease exhibited higher basal miRNAs levels than those with higher stages. The difference was most evident for miR-155 and miR-21 (P=0.05). From the initial to the fourth cycle of chemotherapy, the miRNA levels changed substantially. In samples in which the miRNA levels generally declined, a marked decrease (≤15,500-fold) was evident for the abundant miRNAs. Notably, the occurrence of a decrease in miRNA levels was more frequent in patients with smaller tumor sizes (P<0.05 for miR-21 and miR-195). This proof-of-concept study provides evidence that highly expressed miRNAs are affected most frequently by chemotherapy, particularly in patients with early stage tumors. This information may be valuable in assessing the response of the patients to therapy.

## Introduction

Breast cancer (BC) is the most frequently diagnosed cancer and the leading cause of cancer-related mortality among females worldwide (1). It is estimated that one in eight females will develop BC in their lifetime. Staging in BC is based on tumor size, nodal involvement and distant metastasis, similar to other solid tumors. However, clinical staging is not the only relevant factor in the management of BC. Gene expression profiling studies have revealed that BC consists of heterogeneous subtypes that show substantial variation in their molecular and clinical characteristics (2–4). Clinically, this heterogeneous disease is categorized into the three following therapeutic groups (5): Estrogen receptor (ER)-positive, human epidermal growth factor receptor 2 (*HER2*)-amplified and triple-negative BC [lacking ER, progesterone receptor (PR) and *HER2*]. This high heterogeneity is associated with significant challenges in BC management, and these molecular features are key factors in the response to therapy and disease outcome.

Chemotherapy is an important modality in the treatment of patients with BC. Systemic therapy improves the disease-free survival of patients with BC, but does not lead to remission in the majority of patients with advanced or metastatic disease (6). The resistance to chemotherapeutic agents commonly results in the subsequent recurrence and metastasis of cancer cells. Currently, there is no sensitive and specific biomarker to predict the response to chemotherapy in BC (7).

Extensive studies over the last decade have indicated that microRNAs (miRNA/miRs) are important regulators of numerous cellular processes. Widespread miRNA deregulation in cancer has resulted in the identification of their roles in oncogenesis and tumor-suppression, and has indicated their involvement in cancer initiation, progression and metastasis (8). A number of oncogenic miRNAs have been identified that are associated with BC (9). In recent years, circulating miRNAs have drawn wide interest due to their potential as cancer biomarkers. Several studies have indicated the potential of circulating miRNAs as predictive biomarkers for the early detection and prognosis of cancer (10,11). Although it has been suggested that circulating miRNAs may be associated with the response to treatment, only a few studies have analyzed the predictive value of the pretreatment levels of circulating miRNAs as a marker of chemoresistance in BC (12–14). In concordance with a recent study (15), in early unpublished experiments we determined a number of BC-associated miRNAs, which are at relatively low (let-7, miR-10b, miR-34, miR-155 and miR-200c) or abundant (miR-21, miR-195 and miR-221) levels in the blood plasma of patients with BC. The aim of the present study was to investigate the changes in the plasma levels of these miRNA molecules in response to treatment and their association with the clinicopathological parameters.

## Materials and methods

### Patients

This study included a cohort of 25 female patients with invasive ductal carcinoma of the breast. The patients had been diagnosed with primary operable (T2-3N1) or locally advanced (T1-3N2 or T4Nx) disease. The patient characteristics are shown in Table I. The inclusion criteria for enrolment in the study were normal bone marrow, no contraindication for chemotherapy, no cardiac, renal or hepatic limitations and the provision of informed consent. Peripheral blood was obtained prior to treatment and at the end of the fourth cycle of neoadjuvant chemotherapy (four cycles of 90 mg/m^2^ epirubicin and 600 mg/m^2^ cyclophosphamide). The study was approved by the Istanbul Medical Faculty Local Ethics Committee of Istanbul University (Istanbul, Turkey) and the experiments were undertaken with the understanding and written consent of each subject. The study conforms with The Code of Ethics of the World Medical Association (Declaration of Helsinki).

### miRNA quantification in plasma

Cell-free RNA molecules were extracted from the plasma using TRIzol reagent (Applichem, Düren, Germany). Briefly, 200 μl of the plasma sample was mixed with 800 μl TRIzol reagent, and homogenized. The mixture was added to 200 μl chloroform and incubated on ice for 5 min. Following centrifugation at 12,550 × g for 5 min, the upper RNA phase was transferred into 550 μl propanol containing tubes. Following centrifugation at 12,550 × g, the RNA containing pellet was washed with 75% ethanol, air-dried and dissolved in polymerase chain reaction (PCR)-grade water.

cDNA was synthesized using the miScript Reverse Transcription kit (Qiagen, Valencia, CA, USA) according to the manufacturer’s instructions. The kit includes a poly-A polymerase and a reverse transcriptase. The first step adds a poly-A tail to the 3′-end of the small RNA molecules, while in the second step, the reverse transcriptase converts RNA to cDNA. To quantitate the miRNAs, miScript Primer assays (Qiagen) were used, which include a universal primer specific to the poly-A tail and a miRNA-specific primer. SYBR Green (Qiagen) was used as the florescent molecule. The amplified PCR product had a size of ~80 bp. The small RNA molecule, RNU1A (Qiagen), was used as a control.

Quantitative PCR was performed using LightCycler Instrument 480 (Roche Diagnostics, Mannheim, Germany). The PCR program included a fast start step of 10 min at 95°C followed by 45 cycles of amplification. Each cycle consisted of denaturation at 95°C for 10 sec, annealing at 60°C for 10 sec and elongation at 70°C for 10 sec.

As the reference molecule, miR-205 was used, which showed relatively stable expression [cross-over threshold (Ct) of <30)] throughout all samples and was not significantly affected by the therapy. For quantitation, the comparative ΔCt method was used. Serial dilutions of cDNA were generated using primers for the internal control of miR-205, which exhibited a linear decrease in expression (correlation coefficient=0.99). These dilution series were co-amplified in each PCR session, and the expression values of the internal control and miRNAs from a given sample were derived from the Ct values using the dilution standard. The quantitative experiments were performed twice and the mean values were calculated.

### Statistical analyses

The Pearson correlation test was used to evaluate the correlation between the individual miRNA molecules. Mann-Whitney U and Wilcoxon tests were utilized to assess the univariate differences between the pretreatment plasma levels and clinicopathological parameters, or between the changes during neoadjuvant chemotherapy and clinicopathological parameters. Statistical analyses were conducted with SPSS version 16.0 (SPSS, Inc., Chicago, IL, USA). P<0.05 was used to indicate a statistically significant difference.

## Results

### Pretreatment plasma levels of miRNAs and their association with clinicopathological parameters

**Figure 1:**
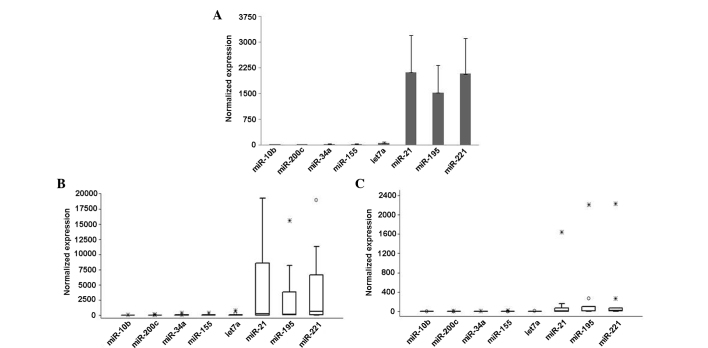
Expression of miRNAs in the blood of breast cancer (BC) patients. Plasma levels of the miRNAs were measured by quantitative polymerase chain reaction (PCR) using miR-205 as the internal control. Data shown are normalized levels in (A) the whole patient group, (B) stage II patients and (C) stage III–IV patients. (A) Data are presented as the mean ± standard error; (B and C) box plots show maximum, median and percentiles. Circles and asterisks represent outlier values. miRNA/miR, microRNA.

miR-21, miR-221 and miR-195 had the highest basal expression levels in the blood plasma, while other miRNAs were expressed at much lower levels (Fig. 1A). The basal plasma levels of eight miRNAs were highly variable between the patients; for example, the relative expression level of miR-221 ranged from 2.3 to 18,900 (median, 63; mean, 2,080). The rates were similar for miR-21 and miR-195. A high correlation was identified between individual miRNAs (P<0.01 for all), with the exception of miR-34, where the correlation with miR-21 (P=0.04) and miR-195 (P=0.06) was somewhat weaker.

The association between the pretreatment miRNA levels and clinicopathological parameters were investigated, and the plasma levels of all miRNAs were observed to be higher in the patients with stage II tumors (n=13) than in those with higher tumor stages (stage III–IV; n=12) (Fig. 1B and C). The difference was most significant for miR-155, miR-21 (both P=0.05) and miR-221 (P=0.07). For the discrimination of ER-positive and -negative cases, the most useful molecule was let-7, with pretreatment levels that were lower in the patients with ER-negative tumors. No associations were observed between the basal miRNA levels and PR status, c-erbB2 levels or Ki67 index.

### Change of the miRNA levels during neoadjuvant chemotherapy and their association with clinicopathological parameters

**Figure 2:**
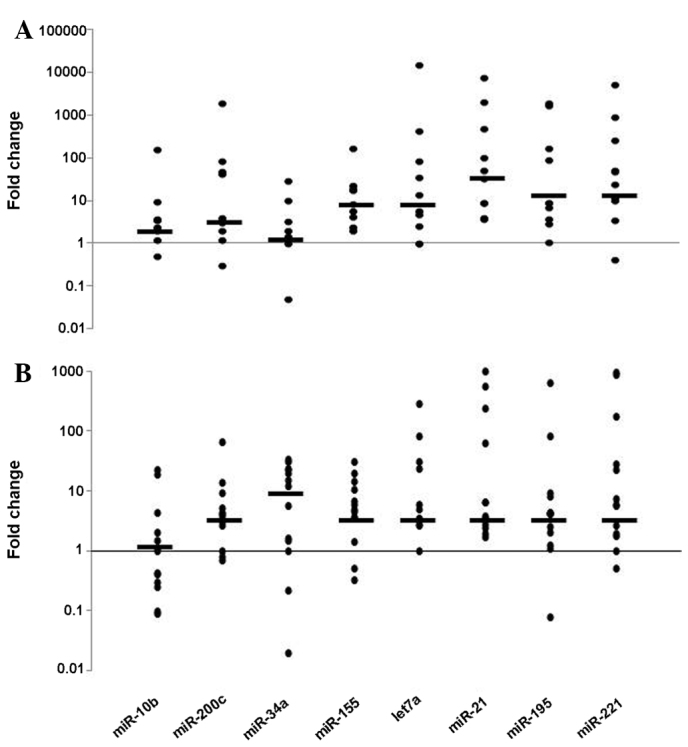
Course of fold-change of miRNAs during neoadjuvant chemotherapy. miRNA levels (A) generally decreased in more than half of patients, and (B) mostly increased in the remaining patients. The axis representing the relative expression is logarithmic and horizontal lines on dots indicate the median values for each molecule. miRNA/miR, microRNA.

When compared with the basal levels, the plasma levels of the majority of the miRNAs at the fourth cycle of neoadjuvant chemotherapy had decreased in half of the patients (n=13; Fig. 2A) whilst they had increased in the other half (n=13; Fig. 2B). The rate of change was highly variable among the miRNA molecules. In general, for the molecules that had high expression levels (such as miR-21, miR-195 and miR-221), the extent of change was also higher among all samples (~500-fold on average). For samples exhibiting an increase following therapy, the highest change was 980-fold. The samples in which the miRNA levels declined exhibited a much more pronounced change (≤15,500-fold). Notably, decreasing levels were more frequent in patients with early-stage disease, and the change was statistically significant for miR-21 and miR-195 (P<0.05). In patients with a lower Ki-67 index, decreasing levels were more frequently observed for the let-7 molecule (P=0.02), while the change was not significant for other molecules. When the clinical pathological response was categorized as ‘adequate’ versus ‘complete’, no significant differences were observed between the pre- and post-treatment levels.

## Discussion

The present study was conducted to investigate the manner in which systemic therapy in the neoadjuvant setting affects circulating miRNAs of low or high levels in patients with BC. Considering the pretreatment levels of eight miRNAs, miR-21, miR-221 and miR-195 had the highest expression levels. This is in concordance with previous studies describing high levels of these miRNAs in the blood circulation of patients with BC (12,16,17). As expected, the plasma levels of the miRNAs were highly variable among the patients; those with stage II tumors exhibited higher plasma levels than those with higher tumor stages. miR-155 was the most distinct molecule for discrimination, however, this finding was not in concordance with a study by Chen *et al* (18), which reported higher miR-155 expression in advanced tumors. Another notable observation in the present study was the lower plasma let-7 levels in patients with ER-negative tumors. Consistent with this finding, previous studies have revealed that let-7 is a negative regulator of ERα signaling (19,20), indicating that plasma let-7 levels may serve as a marker for the ER status.

The effect of chemotherapy on plasma miRNA levels was found to vary between patients in the present study. The extent of change was also highly variable for the individual miRNAs. The change was much more pronounced (≤15,500-fold) for the samples in which the miRNA levels decreased compared with the samples which showed an increase. This may present as a further indication that these miRNAs are highly expressed in breast tumors. Notably, in patients with stage II tumors who had higher miRNA plasma levels than those with advanced stages, the rate of decrease following neoadjuvant chemotherapy was more frequent, which may reflect shrinkage of the tumor. This observation is also consistent with a study indicating that miR-221 induces cell survival and resistance in cancer (21), since a favorable therapy response was observed in all patients. A similar finding during chemotherapy has been described for miR-155 (7). Decreasing levels of let-7 were more frequent in patients with a lower Ki-67 index (P=0.02), while no significant differences were observed among the other molecules. Thus, the let-7 molecule may be a candidate to follow the response of BC patients who have a low proliferation index. In this series, almost all patients had a fair/complete pathological response to therapy, even though certain patients exhibited declining plasma miRNA levels while others exhibited increasing miRNA levels. A previous study by Zhao *et al* (12) reported that patients with varying miR-221 plasma levels exhibited significant differences in the overall response rate, but not in the pathological complete response rate. This indicated that patients with a good pathological response may exhibit varying plasma levels for a given miRNA.

In conclusion, miR-21, miR-195 and miR-221 are highly expressed in the blood circulation of BC patients and are affected most frequently by chemotherapy, particularly in patients with early-stage tumors. This information may be valuable in future studies when assessing the response of patients to therapy. Individual miRNAs, such as let-7, appear to bear potential for use in assessing patients subgroups with given clinicopathological characteristics, such as ER status or Ki-67 index.

## Figures and Tables

**Table I:** Clinicopathological characteristics of the patients.

Characteristics	Value
Age, years	
Median	47.5
Range	36–68
Staging, n	
Stage II	12
Stage III–IV	13
Her-2 status, n	
Positive	12
Negative	13
Estrogen receptor, n	
High positive	12
Low positive	5
Negative	8
Progesterone receptor, n	
High positive	6
Low positive	8
Negative	11
Ki-67 index, n	
High	13
Low	12
Pathological response, n	
Minimal/good	17
Complete	8
